# Chromosomal Abnormalities in Syndromic Orofacial Clefts: Report of Three Children

**DOI:** 10.1155/2018/1928918

**Published:** 2018-09-09

**Authors:** Rathika Damodara Shenoy, Vijaya Shenoy, Vikram Shetty

**Affiliations:** ^1^Department of Pediatrics, K.S. Hegde Medical Academy, Nitte (Deemed to be University), Karnataka, India; ^2^Nitte Meenakshi Institute of Craniofacial Surgery, Nitte (Deemed to be University), Karnataka, India

## Abstract

This case series of three children reports clinical features and chromosomal abnormalities seen in a craniofacial clinic. All presented with orofacial cleft, developmental or intellectual disability, and dysmorphism. Emanuel syndrome or supernumerary der (22)t(11; 22), the prototype of complex small supernumerary marker disorders, was seen in one child. Duplication 4q27q35.2 with concomitant deletion 21q22.2q22.3 and duplication 12p13.33p13.32 with concomitant deletion 18q22.3q23 seen in the remaining two children are not reported in literature. Maternal balanced translocation was established in both of these children.

## 1. Introduction

Orofacial cleft (OFC) is a common congenital anomaly with a prevalence of 1 in 600 live-births. It includes cleft lip, cleft lip with palate (CLP), and cleft palate (CP). Chromosomal etiology accounts for 6% of all children with OFC [1]. In birth cohort studies on clefts the most widely reported chromosomal disorders are trisomies 18 and 13 with poor survival beyond the age of one year [2–4]. Older children with cleft, developmental delay (DD), and minor dysmorphism are encountered in craniofacial clinics. Chromosomal etiology in these children may go unrecognized in infancy due to subtlety in presentation. Chromosomal microarray (CMA) is the first-line investigation of children with congenital anomalies, DD, and intellectual disability [5]. Children with DD and craniofacial defects show significantly higher burden of genomic rearrangements than children with DD and autism or seizures [6]. Array-based techniques have identified candidate chromosomal loci in children with CLP [7]. This series describes the chromosomal abnormalities seen in three children who presented to our craniofacial clinic with DD and dysmorphism in addition to cleft.

## 2. Methods

Genomic DNA was extracted from whole blood treated with EDTA by standard protocol using protein precipitation solution (Qiagen, Germany). DNA concentration and purity were measured by biospectrometer (Eppendorf, Germany). Purified DNA was dissolved with nuclease free water and stored at −20°C until further processing. The CMA was performed with whole genome scanning panel (Illumina HumanCytoSNP-12 Beadchip, USA) according to manufacturer's protocol utilizing 200 ng of DNA per sample in an accredited laboratory. This panel is incorporated with ~300,000 single nucleotide polymorphism probes with dose sensitivity of >800 genes. The resolution was 30kb for copy number variations and higher for regions of known cytogenetic importance. Data output was analyzed using GenomeStudio and KaryoStudio provided by the manufacturer.

Karyotype was performed on metaphase chromosomes obtained from peripheral lymphocytes and twenty Giemsa banded (450-500 level) spreads were studied. The International System for Human Cytogenetic Nomenclature was used for reporting [8]. Snapshots to visualize the genomic rearrangements were done using University of California Santa Cruz (UCSC) browser human assembly GRCh37/hg19, February 2009 (http://genome.ucsc.edu/).

Parental consent for photography and diagnostics was obtained for all the children presented in this series upon approval by the study institute ethics committee.

## 3. Case Presentation

### 3.1. Proband 1

**Figure 1:**
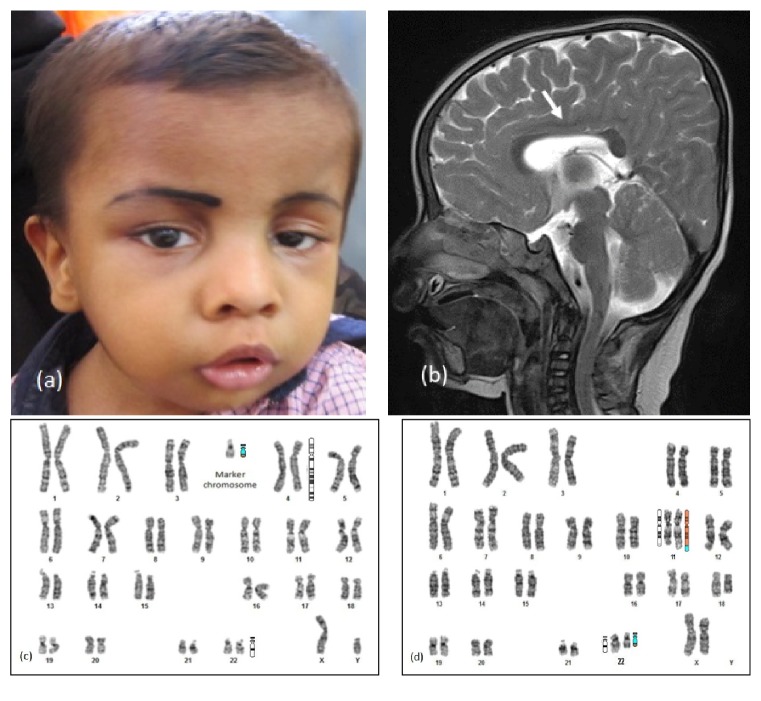
Proband 1 with Emanuel syndrome: (a) phenotype with unilateral left ptosis, microretrognathia, (b) T2 weighted magnetic resonance imaging of brain (sagittal) showing hypoplastic body of corpus callosum (arrow), (c) karyotype and ideogram showing marker chromosome, and (d) karyotype and ideogram of mother showing t(11; 22)(q24; q12).

The infant, first born male, was seen at ten months of age with DD and seizures. There was no parental consanguinity. He had microcephaly and central hypotonia. Dysmorphism included unilateral ptosis, deep-set eyes, low set ears, CP, and micrognathia (Figure 1(a)). Magnetic resonance imaging (MRI) of the brain showed thinning of corpus callosum (Figure 1(b)). Karyotype showed marker chromosome (Figure 1(c)) and CMA partial trisomy of 11q and 22q characteristic of Emanuel syndrome (ES) [9]. Mother had balanced translocation involving chromosomes 11 and 22 (Figure 1(d)).

### 3.2. Proband 2

**Figure 2:**
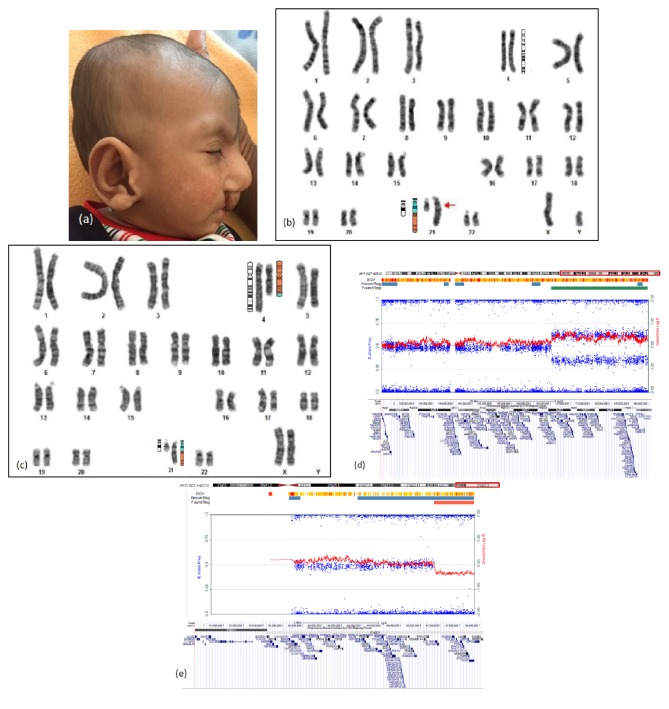
Proband 2: (a) phenotype showing prominent nose, right cleft lip, and abnormal ear, (b) karyotype and ideogram showing derivative chromosome 21, (c) karyotype and ideogram of mother of proband showing t(4; 21)(q27; q22), (d) snapshots of microarray plots (Illumina HumanCytoSNP-12, CA) and coding genes (http://genome.ucsc.edu/) of duplication 4q27q35.2, and (e) snapshots of microarray plots (Illumina HumanCytoSNP-12, CA) and coding genes (http://genome.ucsc.edu/) of deletion 21q22.2q22.3.

This male child born of nonconsanguineous parentage presented with failure to thrive at four months of age. There was sibling death with congenital heart disease. He had microcephaly and spasticity. Dysmorphism included downward slant, prominent nose, ear anomalies, right CLP, retrognathia (Figure 2(a)), and rocker bottom feet. Atrial septal defect and patent ductus arteriosus were visualized on echocardiogram. Evaluation for renal anomalies revealed posterior urethral valve. MRI brain was normal. Karyotype showed derivative chromosome (Figure 2(b)) and CMA showed partial trisomy of 4q and partial monosomy of 21q. Mother had a balanced translocation involving chromosomes 4 and 21 (Figure 2(c)). Snapshots of microarray plots and coding genes in sequential order of dup 4q27q35.2 and del 21q22.2q22.3 regions are shown in Figures 2(d) and 2(e).

### 3.3. Proband 3

**Figure 3:**
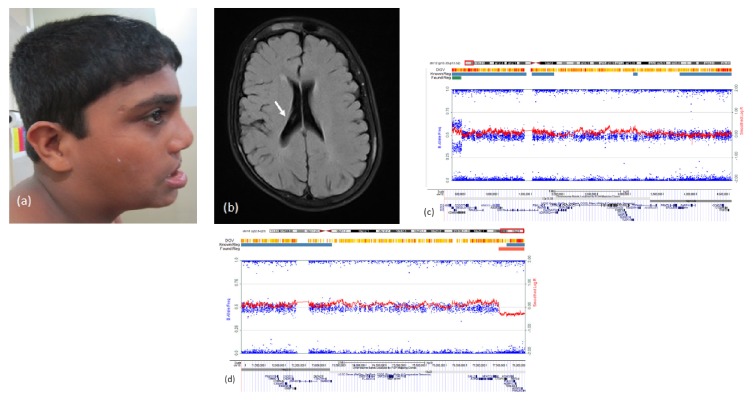
Proband 3: (a) phenotype showing low set ears and everted lower lip, (b) T2 weighted magnetic resonance imaging of brain showing asymmetric lateral ventricle (arrow), (c) snapshots of microarray plots (Illumina HumanCytoSNP-12, CA) and coding genes (http://genome.ucsc.edu/) of duplication 12p13.33p13.32, and (d) snapshots of microarray plots (Illumina HumanCytoSNP-12, CA) and coding genes (http://genome.ucsc.edu/) of deletion 18q22.3q23.

This 9-year-old boy born of nonconsanguineous parentage had intellectual disability with normal neurologic examination. He had hypertelorism, wide eyebrows, narrow nasal root, everted lower lip, short ears, short neck (Figure 3(a)), and operated scar of bilateral cleft lip with alveolus. MRI brain showed asymmetric lateral ventricle (Figure 3(b)). His CMA revealed partial trisomy of 12p and partial monosomy of 18q. Snapshots of microarray plots and coding genes in sequential order of dup 12p13.33p13.32 and del 18q22.3q23 are shown in Figures 3(c) and 3(d). Parental evaluation was normal.

Table 1 gives the molecular karyotype of these children with base pair location, size, number of genes involved, and the critical regions for phenotype correlation.

## 4. Discussion

ES (Proband 1) is the prototype of complex and small supernumerary marker disorders. The most common non-Robertsonian rearrangement in humans is the translocation between chromosomes 11 and 22 as these chromosomes have palindromic AT repeats that are vulnerable to breaks and recombination during meiosis. ES results from 3:1 mal-segregation of usually the maternal balanced carrier state as in our case [9, 10]. The incidence of ES is not known. Features typical of ES were seen in Proband 1. CP is reported in 50% and hypoplastic corpus callosum in 20% among children with ES [11]. However, preauricular pits noted in around 75% were not seen.

Partial trisomy 4q A is usually seen with autosomal monosomy; however, concomitant partial monosomy 21 is not reported in literature. Rinaldi et al. [12] describe a child with congenital heart defect, bilateral hydronephrosis, and partial trisomy of 4q24qter. Renal hypoplasia has been documented by others [13, 14]. Renal anomalies thus seem to be the major system involved in partial terminal 4q trisomy. CLP appears to be rare in this chromosomal abnormality [15]. Developmental or intellectual disability is reported in genomic arrangements of either of the chromosomes 4q and 21q [15, 16]. The breakpoints described with partial monosomy 21q are variable and the phenotype described is mild [16, 17]. Therefore, the features seen in the proband were likely due to partial trisomy 4q.

Partial monosomy of 18q is associated with OFC with 18q22.3q23 as the critical region [18, 19]. Congenital aural atresia described with this partial monosomy was not seen in Proband 3. Partial trisomy of 12p13.3 occurring with partial monosomy 18q is not reported in literature. Parental evaluation was normal suggesting a de novo rearrangement. The wide eyebrows, everted lower lip, short neck, and intellectual disability were features of partial trisomy 12p [20, 21].

To conclude, this series compiles some of the rare and unreported chromosomal abnormalities that include duplication 4q27q35.2 with concomitant deletion 21q22.2q22.3 and duplication 12p13.33p13.32 with concomitant deletion 18q22.3q23. The report also substantiates 4q25q31.3 as a critical locus for renal anomaly and 18q22.3q23 for OFC. In a craniofacial clinic it may be important to identify subtle dysmorphism and DD to establish a chromosomal etiology.

## Figures and Tables

**Table 1:** Molecular karyotype with chromosomal base pair location, size, number of genes, and critical regions in the case series.

**Proband**	**Molecular karyotype**	**Base pair location (mb)**	**Size (mb)**	**Number of genes**	**Critical regions**
1.	arr11q23.3q25 x 3;	116.7 – 134.9	18.2	180	Duplicated material
	arr22q11.1q11.21 x 3	16.1 – 20.7	4.6	65	
2.	arr4q27q35.2 x 3;	121.7 – 190.9	69	226	4q33q34 – Developmental disability and craniofacial dysmorphism 4q26q27 – Cardiac 4q25q31.3 – Urogenital
	arr21q22.2q22.3 x 1	40.8 – 48.1	7.3	105	
3.	arr12p13.33p13.32 x 3;	0.2 – 4.5	4.3	33	18q22.2q23 – Cleft lip palate
	arr18q22.3q23 x 1	70.9 – 78.0	7.1	28	
